# Free Vibration of Functionally Graded Graphene Platelets Reinforced Magnetic Nanocomposite Beams Resting on Elastic Foundation

**DOI:** 10.3390/nano10112193

**Published:** 2020-11-03

**Authors:** Dongying Liu

**Affiliations:** School of Civil Engineering, Guangzhou University, Guangzhou 510006, China; liudy@gzhu.edu.cn

**Keywords:** free vibration, magnetic field, two-dimensional elasticity theory, beams, GPL-reinforced nanocomposites

## Abstract

The vibrational characteristics of multilayer magnetic nanocomposite beams reinforced by graphene nanoplatelets (GPLs) are analytically investigated in this paper. The effects of an elastic foundation are also studied. The material properties of piece-wise GPL-reinforced nanocomposites (GPLRCs) are assumed to be graded in the thickness direction of the beams and can be estimated by using the modified Halpin–Tsai model and rules of mixtures. The two-dimensional elasticity theory is adopted to derive the governing equation combined with the state space method, and the analytical frequency equations for simply supported beams are obtained. In addition, the effects of a magnetic field are involved via Maxwell’s equation, and the corresponding Lorentz forces are considered in this work. Numerical examples are carried out to examine the effects of magnetic fields in various directions, the GPL distribution pattern, the scale parameter and weight function of GPLs, as well as an elastic foundation, on the vibration behaviors of functionally graded (FG)-GPLRC beams.

## 1. Introduction

Graphene-based nanocomposites, a novel kind of promising functionalized nanomaterials, show a wide range of applications in engineering, such as sensors, fuel cells, supercapacitors, and batteries, due to the excellent mechanical, chemical, and physical properties [1,2,3,4]. The recent trend is the addition of graphene nanoplatelets (GPLs) as nano-reinforcements dispersed into a polymer matrix to yield polymer nanocomposites. GPL-reinforced polymer nanocomposites have proven to exhibit better mechanical, thermal, optical, and electrical properties than neat polymer materials [5,6]. Rafiee et al. [7] examined the mechanical properties of epoxy nanocomposites with a low content of graphene platelets, and they compared the reinforced effects with single-walled carbon nanotubes, multi-walled carbon nanotube, and GPLs. They found that at the same weight fraction of nanofillers, the Young’s modulus of graphene nanocomposites was nearly 31% greater than the pristine epoxy as compared to the 3% increase for single-walled carbon nanotubes. Meantime, the tensile strength, fracture toughness, and fatigue resistance were also dramatically promoted. Ji et al. [8] investigated the stiffening effect of graphene sheets dispersed in polymer nanocomposites using the Mori–Tanaka micromechanics model. It was shown that a very low content of graphene sheets significantly enhanced the effective stiffness of the nanocomposites. Chandra et al. [9] proposed a multiscale finite element method to study the natural frequencies and mode shapes of graphene/polymer nanocomposite structures, and it was shown that graphene nanofillers are thought to be a potential replacement for conventional composite fibers such as carbon nanotubes and glass fibers. The superiority of GPLs over the conventional composite fibers in terms of mechanical properties enhancement is related to their high specific surface area, enhanced nanofiller-matrix adhesion, and interlocking, as well as the two-dimensional geometry of GPLs.

Functionally graded materials (FGMs) are a special kind of composites in which the composition and structure gradually change, resulting in corresponding changes in the properties of composites. FGMs have extensively received wide application in almost every discipline of engineering including aerospace, electronics, optics, and civil engineering [10]. Motivated by these engineering applications, FGMs have also attracted intensive research interests. Loy et al. [11] studied the free vibration of FGM plates, in which the volume fraction power-law variation of material properties was used. Chen et al. [12] applied an approximate laminate model to analyze nonhomogeneous hollow circular cylinders. Trinh [13] performed a semianalytical stochastic buckling quantification of porous functionally graded plates. Malikan et al. [14] developed a nonlocal three-dimensional theory of elasticity to examine the buckling of functionally graded porous nanoplates. Malikana and Eremeyev [15,16] proposed a hyperbolic-polynomial higher-order elasticity model to check out the material composition of thick FGM beams and plates.

To entirely utilize the reinforcing efficiency of GPLs, the concept of functionally graded materials (FGMs) is combined with graphene-based nanocomposites [17]. The volume/weight fraction of GPLs varies linearly or nonlinearly through the thickness of the structures, and the mechanical behaviors of the nanocomposites emerge as functional gradations. Yang and his coauthors proposed the piece-wise FG-GPL reinforced models for beams [18,19,20], plates [21,22,23,24,25], and shells [26,27,28], where the isotropic Halpin–Tsai equation was adopted to predict the effective Young’s modulus of the nanocomposites. Following the pioneered works, an extended Halpin–Tsai model was developed for anisotropic graphene-based nanocomposites [29], and the nonlinear bending, nonlinear buckling, and postbuckling of beams [30,31], plates [32,33], and shells [34,35] were investigated in detail based on this model.

In recent years, the demand of magnetic materials is essential for modern industry. In particular, carbon-based composite materials have generated extreme interest, which resulted in findings such as an easier theoretical magnetism prediction and a more likely spin induction [36,37]. Moreover, graphene-based nanocomposites are deemed as a promising s- or p-electron-based magnet [38,39]. Murmu et al. [40] studied the effects of an in-plane magnetic field on the transverse vibration of a magnetically sensitive single-layer graphene sheet using the equivalent nonlocal elasticity approach, and numerical results revealed that the natural frequencies of the graphene sheet were increased by exerting the in-plane magnetic field. Karličić et al. [41] proposed a new type of mass-nanosensor by vibrating graphene sheets within a magnetic field. Li et al. [42] reported the nonlinear dynamic responses of triple-layered graphene sheets under an external magnetic field, which could serve as references in the application of nanocomposites based on laminated graphene sheets. Malikana and his coauthors [43,44] investigated the stability of nanocomposite shell and piezo-magnetoelectric nanoplates in magnetic fields with the aid of the nonlocal strain gradient theory. Sobhy [45] analyzed the buckling and vibration of FG-GPL reinforced aluminum sandwich curved beams exposed to a magnetic field. Mohammadimehr et al. [46] studied the magneto-mechanical vibration of a GPL-reinforced micro-Timoshenko porous beam. In brief, the graphene-based magnets open up new ways to design nanosensors as well as other micro- or nanodevices, and this further highlights the importance of magnetic graphene, since graphene has extraordinary carrier mobility and may provide an easy way to integrate spin and molecular electronics.

With the fast-increasing applications of graphene-based nanocomposites, understanding the mechanical behaviors of such nanocomposite structures in external magnetic fields are of great practical importance. Therefore, the main aim of the present paper is to examine the free vibration of functionally graded GPL reinforced multilayer nanocomposite beams resting on an elastic foundation within in-plane and out-of-plane magnetic fields. The material properties of GPL-reinforced nanocomposites are evaluated by the modified Halpin–Tsai model and the rules of mixtures. Different linear distribution patterns of GPL nanofillers through the thickness of the beams, namely UD, FG-X, FG-O, FG-V and FG-A, are considered in this work. A two-dimensional elasticity is adopted to establish the governing equation with the aid of the state space method, and the analytical solution of vibration frequencies for simply supported beams is obtained. The effects of magnetic fields, GPL weight fraction, GPL distribution pattern, geometry and size of GPLs, as well as the Winkler–Pasternak foundation, on the natural frequencies of functionally graded GPL-reinforced nanocomposites (FG-GPLRC) beams are discussed in detail.

## 2. Theoretical Formulation

**Figure 1:**
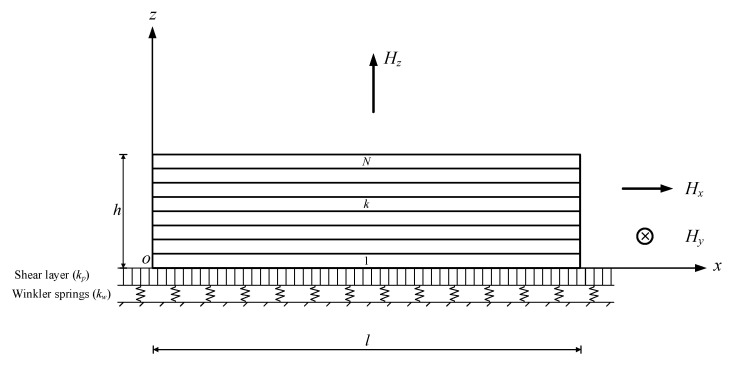
Schematic of a functionally graded graphene nanoplatelet reinforced nanocomposite (FG-GPLRC) beam resting on an elastic foundation in magnetic fields.

The multilayer beam composed of FG-GPLRCs with thickness *h*, unit width, and length *l* is defined in a Cartesian coordinate system (*x*, *z*), where the *x*-axis is located on the bottom surface and the *z*-axis is parallel to the thickness direction, as shown in Figure 1. The beam is rested on a Winkler–Pasternak elastic foundation, with the foundation moduli of *k_w_* and *k_p_*. In-plane magnetic fields $H_{x}$ and $H_{z}$ as well as out-of-plane $H_{y}$, along the positive direction of the coordinate axes, are considered respectively. The volume fractions of GPLs are assumed to be layer-wised varied and thus result in different GPL distribution patterns through the beam thickness. The nonuniform GPL distribution patterns give rise to the functionally graded behaviors in the thickness direction of the beam. To yield a direct and meaningful comparison, the total content of GPLs remains identical for various GPL distribution patterns. The nanocomposite beam is composed of *N* layers with an identical sublayer thickness $h_{N}=h/N$. In each individual layer, the GPLs are uniformly dispersed with a random orientation in the polymer matrix. Hence, each individual layer can be dealt with as an isotropic homogeneous material.

### 2.1. Micromechanics Model of the FG-GPLRCs

**Figure 2:**
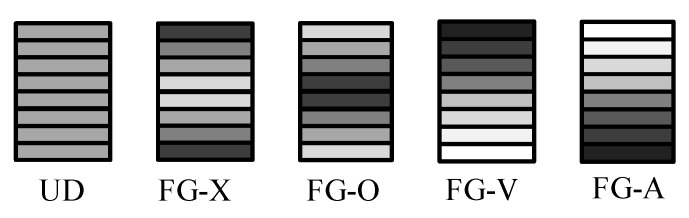
Schematic diagrams of the GPL distribution patterns.

In present, five typical linear distribution patterns of GPLs as shown in Figure 2, namely UD, FG-X, FG-O, FG-V, and FG-A, are to be examined.

The GPL volume fraction $V_{\mathrm{GPL}}$ of an arbitrary layer, say the *j*-th layer (j=1,2,⋯,N), can be described as follows:

(1) $$\begin{array}{ll} \mathrm{UD}: & V_{\mathrm{GPL}}\left(j\right)=V_{\mathrm{GPL}}^{*},\\ \mathrm{FG}-X: & V_{\mathrm{GPL}}\left(j\right)=2V_{\mathrm{GPL}}^{*}\left(\left|2j-N-1\right|/N\right),\\ \mathrm{FG}-O: & V_{\mathrm{GPL}}\left(j\right)=2V_{\mathrm{GPL}}^{*}\left(1-\left|N-2j+1\right|/N\right),\\ \mathrm{FG}-V: & V_{\mathrm{GPL}}\left(j\right)=V_{\mathrm{GPL}}^{*}\left(\left(2j-1\right)/N\right),\\ \mathrm{FG}-A: & V_{\mathrm{GPL}}\left(j\right)=V_{\mathrm{GPL}}^{*}\left(\left(2\left(N-j\right)+1\right)/N\right), \end{array}$$

where $V_{\mathrm{GPL}}^{*}$ is the total volume fraction of GPLs, and can be determined by

(2) $$V_{\mathrm{GPL}}^{*}=\frac{W_{\mathrm{GPL}}}{W_{\mathrm{GPL}}+\left(\rho_{\mathrm{GPL}}/\rho_{M}\right)\left(1-W_{\mathrm{GPL}}\right)}$$

in which $W_{\mathrm{GPL}}$ is the total weight fraction of GPLs, and $\rho_{\mathrm{GPL}}$ and $\rho_{M}$ are the mass densities of GPLs and the polymer matrix, respectively. It can be seen that both the top and bottom surfaces are GPL-rich for the FG-X pattern, while the midplane of the plate is GPLs rich for FG-O. The GPL volume fraction increases monotonically from the bottom surface to the top surface for FG-V, while it reverses for FG-A. As a special case, UD corresponds to a homogeneous beam with uniform GPL distribution.

It has been well demonstrated that the Halpin–Tsai micromechanics model is one of the most common methods to evaluate the effective Young’s modulus of two-phase nanocomposite materials. The accuracy of this model was experimentally validated for the nanocomposites composed of epoxy and the low content of GPLs [7]. Accordingly, the effective elastic modulus of GPL-reinforced nanocomposites can be approximated as follows [17]:

(3) $$E=\frac{3}{8}\frac{1+\xi_{l}\eta_{l}V_{\mathrm{GPL}}}{1-\eta_{l}V_{\mathrm{GPL}}}\times E_{M}+\frac{5}{8}\frac{1+\xi_{w}\eta_{w}V_{\mathrm{GPL}}}{1-\eta_{w}V_{\mathrm{GPL}}}\times E_{M}$$

where the parameters $\eta_{l}$ and $\eta_{w}$ are expressed as

(4) $$\eta_{l}=\frac{\left(E_{\mathrm{GPL}}/E_{M}\right)-1}{\left(E_{\mathrm{GPL}}/E_{M}\right)+\xi_{l}},\;\;\eta_{w}=\frac{\left(E_{\mathrm{GPL}}/E_{M}\right)-1}{\left(E_{\mathrm{GPL}}/E_{M}\right)+\xi_{w}}$$

in which $E_{\mathrm{GPL}}$ and $E_{M}$ are the elastic moduli of GPLs and matrix, respectively. Meanwhile, $\xi_{l}$ and $\xi_{w}$ can be determined by

(5) $$\xi_{l}=2l_{\mathrm{GPL}}/h_{\mathrm{GPL}},\;\;\xi_{w}=2w_{\mathrm{GPL}}/h_{\mathrm{GPL}}$$

which represents the effect of the geometry and size of GPL nanofillers with $l_{\mathrm{GPL}}$, $w_{\mathrm{GPL}}$, and $h_{\mathrm{GPL}}$ being the length, width, and thickness of GPLs, respectively.

By employing the rule of mixtures, the effective mass density ρ, Poisson’s ratio ν, and the magnetic field permeability η of the GPL-reinforced nanocomposites can be calculated as follows [28,47]:

(6) $$\begin{array}{l} \rho =\rho_{\mathrm{GPL}}V_{\mathrm{GPL}}+\rho_{M}\left(1-V_{\mathrm{GPL}}\right)\\ \nu =\nu_{\mathrm{GPL}}V_{\mathrm{GPL}}+\nu_{M}\left(1-V_{\mathrm{GPL}}\right)\\ \eta =\eta_{\mathrm{GPL}}V_{\mathrm{GPL}}+\eta_{M}\left(1-V_{\mathrm{GPL}}\right) \end{array}$$

where $\nu_{\mathrm{GPL}}$ and $\nu_{M}$ are the Poisson’s ratios, and $\eta_{\mathrm{GPL}}$ and $\eta_{M}$ are the corresponding magnetic field permeabilities of GPLs and the matrix, respectively.

### 2.2. Maxwell’s Relation

According to the classical electromagnetic theory, the current density **J**, distributing vector of magnetic field **h**, strength vectors of the electric field **e**, and magnetic field permeability *η* can be related by Maxwell’s equations in differential form and can be expressed as follows [36]:

(7) $$J=\nabla \times h,\;\nabla \times e=-\eta \frac{\partial h}{\partial t},\;\nabla \cdot h=0$$

where the vectors of distributing magnetic field **h** and the electric field **e** can be determined as

(8) $$h=\nabla \times \left(U\times H\right),\;\;e=-\eta \left(\frac{\partial U}{\partial t}\times H\right)$$

in which $\nabla =\frac{\partial }{\partial x}i+\frac{\partial }{\partial y}j+\frac{\partial }{\partial z}k$ is the Hamilton operator; U=ui+vj+wk is the displacement vector; and $H=\left(H_{x},H_{y},H_{z}\right)$ is the magnetic field vector where $H_{x}$ and $H_{z}$ are components of the in-plane magnetic field, and $H_{y}$ the out-of-plane magnetic field.

By using Equations (7) and (8), the Lorentz force vector induced by the magnetic field can be obtained as

(9) f=η(J×H)

Further, for the beam considered herein, there are only the longitudinal and transverse displacements *u* and *w*, and thus the *x*-, *y*-, and *z*-direction components of Lorentz force can be expressed as

(10) $$\begin{array}{l} f_{x}=\eta \left[H_{y}^{2}\left(\frac{\partial^{2}w}{\partial x\partial z}+\frac{\partial^{2}u}{\partial x^{2}}\right)+H_{z}^{2}\left(\frac{\partial^{2}u}{\partial z^{2}}+\frac{\partial^{2}u}{\partial x^{2}}\right)\right]\\ f_{y}=0\\ f_{z}=\eta H_{x}^{2}\left(\frac{\partial^{2}w}{\partial x^{2}}+\frac{\partial^{2}w}{\partial z^{2}}\right) \end{array}$$

### 2.3. Structural Problem Formulation

The electromagnetic dynamic equations for an arbitrary layer, say the *j*-th layer, of the FG-GPLRC beam, neglecting the body force, are expressed as follows [48]:

(11) $$\begin{array}{l} \frac{\partial \sigma_{x}}{\partial x}+\frac{\partial \tau_{xz}}{\partial z}+f_{x}=\rho \frac{\partial^{2}u}{\partial t^{2}}\\ \frac{\partial \tau_{xz}}{\partial x}+\frac{\partial \sigma_{z}}{\partial z}+f_{z}=\rho \frac{\partial^{2}w}{\partial t^{2}} \end{array}$$

where $\sigma_{x}$, $\sigma_{z}$, and $\tau_{xz}$ are the normal and shear stress components, respectively, and $f_{x}$ and $f_{z}$ are the Lorentz forces as given in Equation (10).

The constitutive equations of the FG-GPLRC beam in the state of plane stress can be read as

(12) $$\varepsilon_{x}=\frac{1}{E}\left(\sigma_{x}-\nu \sigma_{z}\right),\;\;\varepsilon_{z}=\frac{1}{E}\left(\sigma_{z}-\nu \sigma_{x}\right),\;\;\gamma_{xz}=\frac{2\left(1+\nu \right)}{E}\tau_{xz}$$

where *E* and ν are the Young’s modulus and Poisson’s ratio of the nanocomposites, which can be determined by Equations (3) and (6), respectively. Normal and shear strain components $\varepsilon_{x}$, $\varepsilon_{z}$, and $\gamma_{xz}$ can be rendered in terms of displacements through geometrical relation as

(13) $$\varepsilon_{x}=\frac{\partial u}{\partial x},\;\;\varepsilon_{z}=\frac{\partial w}{\partial z},\;\;\gamma_{xz}=\frac{\partial u}{\partial z}+\frac{\partial w}{\partial x}$$

The substituting of Equation (13) into Equation (12) yields

(14) $$\frac{\partial u}{\partial x}=\frac{1}{E}\left(\sigma_{x}-\nu \sigma_{z}\right),\;\;\frac{\partial w}{\partial z}=\frac{1}{E}\left(\sigma_{z}-\nu \sigma_{x}\right),\;\;\frac{\partial u}{\partial z}+\frac{\partial w}{\partial x}=\frac{2\left(1+\nu \right)}{E}\tau_{xz}$$

Now, Equations (11) and (14) indicate the governing equations in terms of three stresses, namely $\sigma_{x}$, $\sigma_{z}$, and $\tau_{xz}$, and the two displacements, *u* and *w*.

The FG-GPLRC beam with the simple supports at the two ends is considered herein, which gives

(15) $$\sigma_{x}=w=0,\;\;\left(x=0,\;l\right)$$

The beam resting on a Winkler–Pasternak elastic foundation holds:

(16) $$\sigma_{z}\left(x,0\right)=k_{w}w\left(x,0\right)-k_{p}\frac{\partial^{2}w\left(x,0\right)}{\partial x^{2}}$$

For the beam described above, it can be assumed as follows [48,49]:

(17) $$\left\{\begin{array}{c} \sigma_{z}\\ u\\ w\\ \tau_{xz} \end{array}\right\}=\sum_{n=0}^{\infty }\left\{\begin{array}{c} c_{0}\sigma_{\zeta n}\left(\zeta \right)\sin\left(n\pi \xi \right)\\ hu_{n}\left(\zeta \right)\cos\left(n\pi \xi \right)\\ hw_{n}\left(\zeta \right)\sin\left(n\pi \xi \right)\\ c_{0}\tau_{n}\left(\zeta \right)\cos\left(n\pi \xi \right) \end{array}\right\}e^{i\omega t}$$

where ξ=x/l and ζ=z/h are the nondimensional coordinates, *n* the half-wave number, ω the circular frequency, $i=\sqrt{-1}$ the unit of imaginary, and $c_{0}$ the reference Young’s modulus.

For the ease of establishing the governing equations, the magnetic fields along the *x*, *y*, and *z* directions are discussed separately. For the case of the longitudinal magnetic field $H_{x}$, the corresponding Lorentz forces are

(18) $$\begin{array}{l} f_{x}=f_{y}=0\\ f_{z}=\eta H_{x}^{2}\left(\frac{\partial^{2}w}{\partial x^{2}}+\frac{\partial^{2}w}{\partial z^{2}}\right) \end{array}$$

Substituting (17) into Equations (12), (11) and (18) gives the following state equation in terms of a matrix form as

(19) $$\frac{\partial }{\partial \zeta }\delta_{n}=A_{n}\delta_{n}$$

where $\delta_{n}={\left[\begin{array}{cccc} \sigma_{\zeta n} & u_{n} & w_{n} & \tau_{n} \end{array}\right]}^{T}$ is referred to as the state vector with the superscript T, indicating the transpose of a vector, and the coefficient matrix **A***_n_* is

(20) $$A_{n}=\left[\begin{array}{cccc} 0 & 0 & \bar{\eta }M_{x}\left(1+\frac{{\bar{c}}_{13}}{{\bar{c}}_{33}}\right){\left(\lambda n\pi \right)}^{2}-\left(1-\frac{\bar{\eta }M_{x}}{{\bar{c}}_{33}}\right)\bar{\rho }\Omega^{2} & \left[1-\frac{\bar{\eta }M_{x}}{{\bar{c}}_{33}}\left(1+\frac{{\bar{c}}_{13}}{{\bar{c}}_{55}}\right)\right]\lambda n\pi \\ 0 & 0 & -\lambda n\pi & \frac{1}{{\bar{c}}_{55}}\\ \frac{1}{{\bar{c}}_{33}} & \frac{{\bar{c}}_{13}}{{\bar{c}}_{33}}\lambda n\pi & 0 & 0\\ -\frac{{\bar{c}}_{13}}{{\bar{c}}_{33}}\lambda n\pi & \bar{k}{\left(\lambda n\pi \right)}^{2}-\bar{\rho }\Omega^{2} & 0 & 0 \end{array}\right]$$

in which ${\bar{c}}_{ij}=c_{ij}/c_{0}$, $\bar{k}={\bar{c}}_{11}-{\bar{c}}_{13}^{2}/{\bar{c}}_{33}$, $\bar{\eta }=\eta /\eta_{0}$, and $\bar{\rho }=\rho /\rho_{0}$ are dimensionless parameters with $\eta_{0}$ and $\rho_{0}$ representing the reference magnetic permeability and reference mass density, respectively; $\Omega =\omega h\sqrt{\rho_{0}/c_{0}}$ is the dimensionless natural frequency; and $M_{x}=\eta_{0}H_{x}^{2}/c_{0}$ is the *x*-direction magnetic parameter with λ=h/l being the aspect ratio of the beam.

Similarly, the Lorentz forces induced by the *y*-direction magnetic field turn out to be

(21) $$\begin{array}{l} f_{x}=\eta H_{y}^{2}\left(\frac{\partial^{2}w}{\partial x\partial z}+\frac{\partial^{2}u}{\partial x^{2}}\right)\\ f_{y}=f_{z}=0 \end{array}$$

and the corresponding coefficient matrix **A***_n_* of the state space equation is

(22) $$A_{n}=\left[\begin{array}{cccc} 0 & 0 & -\bar{\rho }\Omega^{2} & \lambda n\pi \\ 0 & 0 & -\lambda n\pi & \frac{1}{{\bar{c}}_{55}}\\ \frac{1}{{\bar{c}}_{33}} & \frac{{\bar{c}}_{13}}{{\bar{c}}_{33}}\lambda n\pi & 0 & 0\\ -\left(\frac{{\bar{c}}_{13}+\bar{\eta }M_{y}}{{\bar{c}}_{33}}\right)\lambda n\pi & \left[\bar{k}+\bar{\eta }M_{y}\left(1-\frac{{\bar{c}}_{13}}{{\bar{c}}_{33}}\right)\right]{\left(\lambda n\pi \right)}^{2}-\bar{\rho }\Omega^{2} & 0 & 0 \end{array}\right]$$

in which $M_{y}=\eta_{0}H_{y}^{2}/c_{0}$ is the *y*-direction dimensionless magnetic parameter.

The Lorentz forces induced by the *z*-direction magnetic field are

(23) $$\begin{array}{l} f_{x}=\eta H_{z}^{2}\left(\frac{\partial^{2}u}{\partial z^{2}}+\frac{\partial^{2}u}{\partial x^{2}}\right)\\ f_{y}=f_{z}=0 \end{array}$$

and the corresponding coefficient matrix **A***_n_* of the state space equation is

(24) $$A_{n}=\left[\begin{array}{cccc} 0 & 0 & -\bar{\rho }\Omega^{2} & \lambda n\pi \\ 0 & 0 & -\lambda n\pi & \frac{1}{{\bar{c}}_{55}}\\ \frac{1}{{\bar{c}}_{33}} & \frac{{\bar{c}}_{13}}{{\bar{c}}_{33}}\lambda n\pi & 0 & 0\\ \frac{{\bar{c}}_{55}\left(\bar{\eta }M_{z}-{\bar{c}}_{13}\right)}{{\bar{c}}_{33}\left(\bar{\eta }M_{z}+{\bar{c}}_{55}\right)}\lambda n\pi & \frac{{\bar{c}}_{55}}{{\bar{c}}_{55}+\bar{\eta }M_{z}}\left[\left(\bar{k}+\bar{\eta }M_{z}\left(\frac{{\bar{c}}_{13}}{{\bar{c}}_{33}}+1\right)\right){\left(\lambda n\pi \right)}^{2}-\bar{\rho }\Omega^{2}\right] & 0 & 0 \end{array}\right]$$

in which $M_{z}=\eta_{0}H_{z}^{2}/c_{0}$ is the dimensionless magnetic field along the *y*-direction.

Thus, the state space equations for the *j*-th layer of the FG-GPLRC beam in the magnetic fields along the *x*, *y*, and *z* directions are established as given in a unified matrix form of Equation (19), and the corresponding coefficient matrices **A***_n_* are defined in Equations (20), (22) and (24), respectively. It should be noted that when the magnetic field vanishes, all the coefficient matrixes are identical and can be degenerated into the one for elastic beams in Reference [48].

According to the matrix theory, the general solution to Equation (19) is

(25) $$\delta_{n}^{\left(j\right)}\left(\zeta \right)=\exp\left[\left(\zeta -\zeta_{j-1}\right)A_{n}^{\left(j\right)}\right]\delta_{n}^{\left(j\right)}\left(\zeta_{j-1}\right),\;\;\left(\zeta_{j-1}\leq \zeta \leq \zeta_{j}\right)$$

where the superscript *j* represents the *j*-th layer of the multilayer beam, and $\zeta_{j-1}=\left(j-1\right)/N$ and $\zeta_{j}=j/N$ are the dimensionless coordinates of the bottom and top surfaces of the *j*-th layer of the beam, respectively.

The state space vector for the top surface of the *j*-th layer can be obtained by setting $\zeta =\zeta_{j}$ into Equation (25), which gives

(26) $$\delta_{n}^{\left(j\right)}\left(\zeta_{j}\right)=\exp\left(A_{n}^{\left(j\right)}/N\right)\delta_{n}^{\left(j\right)}\left(\zeta_{j-1}\right),\;\;\left(j=1,2,\cdots ,N\right)$$

By imposing the continuity conditions at the interface of two arbitrary adjacent layers into Equation (26) through all the *N* layers, the following relation is obtained:

(27) $$\delta_{1}=T\delta_{0}$$

where $\delta_{1}=\delta_{n}^{\left(N\right)}\left(1\right)$ and $\delta_{0}=\delta_{n}^{\left(1\right)}\left(0\right)$ are the state vectors at the top and bottom surfaces of the beam, respectively, and the global transfer matrix is

(28) $$T=T^{\left(N\right)}\cdots T^{\left(j+1\right)}T^{\left(j\right)}\cdots T^{\left(1\right)}=\prod_{j=N}^{1}\exp\left(A_{n}^{\left(j\right)}/N\right)$$

The global matrix **T** in Equation (28) can be rewritten into a partitioning matrix as

(29) $$\left\{\begin{array}{c} \sigma_{\zeta n}\left(1\right)\\ u_{n}\left(1\right)\\ w_{n}\left(1\right)\\ \tau_{n}\left(1\right) \end{array}\right\}=\left[\begin{array}{cccc} T_{11} & T_{12} & T_{13} & T_{14}\\ T_{21} & T_{22} & T_{23} & T_{24}\\ T_{31} & T_{32} & T_{33} & T_{34}\\ T_{41} & T_{42} & T_{43} & T_{44} \end{array}\right]\left\{\begin{array}{c} \sigma_{\zeta n}\left(0\right)\\ u_{n}\left(0\right)\\ w_{n}\left(0\right)\\ \tau_{n}\left(0\right) \end{array}\right\}$$

The lateral boundary conditions in Equation (16) can be re-expressed in terms of nondimensional quantities by using Equation (17) as

(30) $$\sigma_{\zeta n}\left(0\right)=k_{n}w_{n}\left(0\right)$$

where $k_{n}=\frac{\lambda^{4}}{12}\left[K_{w}+{\left(n\pi \right)}^{2}K_{p}\right]$, in which $K_{w}=k_{w}l^{4}/c_{0}I$ and $K_{p}=k_{p}l^{2}/c_{0}I$ are the nondimensional moduli of the elastic foundation, and *I* is the second moment of the cross-sectional area.

The substitution of Equation (30) into (29) yields

(31) $$\left\{\begin{array}{c} \sigma_{\zeta n}\left(1\right)\\ u_{n}\left(1\right)\\ w_{n}\left(1\right)\\ \tau_{n}\left(1\right) \end{array}\right\}=\left[\begin{array}{lll} T_{12} & k_{n}T_{11}+T_{13} & T_{14}\\ T_{22} & k_{n}T_{21}+T_{23} & T_{24}\\ T_{32} & k_{n}T_{31}+T_{33} & T_{34}\\ T_{42} & k_{n}T_{41}+T_{43} & T_{44} \end{array}\right]\left\{\begin{array}{c} u_{n}\left(0\right)\\ w_{n}\left(0\right)\\ \tau_{n}\left(0\right) \end{array}\right\}$$

For free vibration, the top and bottom stress components are known as

(32) $$\sigma_{\zeta n}\left(1\right)=0,\;\;\tau_{n}\left(1\right)=\tau_{n}\left(0\right)=0$$

Substitution of Equation (32) into Equation (31) and elimination of $u_{n}\left(1\right)$ and $w_{n}\left(1\right)$ result in

(33) $$\left[\begin{array}{cc} T_{12} & k_{n}T_{11}+T_{13}\\ T_{42} & k_{n}T_{41}+T_{43} \end{array}\right]\left\{\begin{array}{c} u_{n}\left(0\right)\\ w_{n}\left(0\right) \end{array}\right\}=0$$

For the nontrial solution of Equation (33), the vanishing of the determinant of the coefficient matrix gives

(34) $$\left|\begin{array}{cc} T_{12} & k_{n}T_{11}+T_{13}\\ T_{42} & k_{n}T_{41}+T_{43} \end{array}\right|=0$$

which is the frequency equation about Ω and can be solved numerically to obtain the natural frequencies of the FG-GPLRC beams.

## 3. Results and Discussion

### 3.1. Validation of the Present Method

In order to validate the present method, several numerical examples are carried out for comparisons. Firstly, the natural frequency parameter $\varpi =\sqrt[{4}]{\rho Al^{4}\omega^{2}/EI}$ was computed for the isotropic homogeneous beam resting on elastic foundations. The results for beams with different aspect ratios and different foundation parameters *K_w_* and *K_p_* are compared to those reported in Ref. [48] in Table 1. It is seen that the present numerical results agree well with those reported in the literature. Secondly, the fundamental frequency parameter ϖ of an FGM beam with an elastic foundation is listed in Table 2, and these values are compared to those obtained by Ying et al. [49]. It should be noted that all elastic constants and mass density of the FGM beam are assumed to vary exponentially through the beam thickness, and the layer-wise mode (N=20) was adopted to approximate the FGMs. Excellent agreement is again observed, and hence the present method is verified.

### 3.2. Free Vibration of Epoxy/GPLs Beams in Magnetic Fields

In what follows, detailed parametric studies were carried out to examine the free vibration of the FG-GPLRC beams resting on an elastic foundation in magnetic fields. Herein, the epoxy as the matrix phase and GPLs as the reinforced phase are selected as numerical examples, and the corresponding material constants are listed in Table 3.

Unless otherwise stating, the geometry and size of the GPL nanofillers are respectively $l_{\mathrm{GPL}}=2.5\;\mu m$, $w_{\mathrm{GPL}}=1.5\;\mu m$, and $h_{\mathrm{GPL}}=1.5\;\mathrm{nm}$, and the weight fraction of GPLs is fixed at $W_{\mathrm{GPL}}=1\%$. The ideal graphene is intrinsically nonmagnetic and lacks localized magnetic moments due to a delocalized π-bonding network [50]. Therefore, the magnetic field permeability of GPLs is assumed to be zero in the present discussion. Although the intrinsic graphene cannot be affected by the magnetic field, the additions of GPLs into a magnetic polymer matrix can significantly improve the mechanical properties of the nanocomposites. Therefore, the addition of GPLs into a magnetic matrix can meaningfully affect the dynamical behaviors of the nanocomposite beams in magnetic fields. Moreover, it should be pointed out that the unusual positive magnetic signals (paramagnetic and/or ferromagnetic) have been experimentally reported for synthesizing magnetic graphene in recent years [50]. In addition, the reference physical quantities $c_{0}$, $\rho_{0}$, and $\eta_{0}$ are chosen as the values of the epoxy matrix.

**Figure 3:**
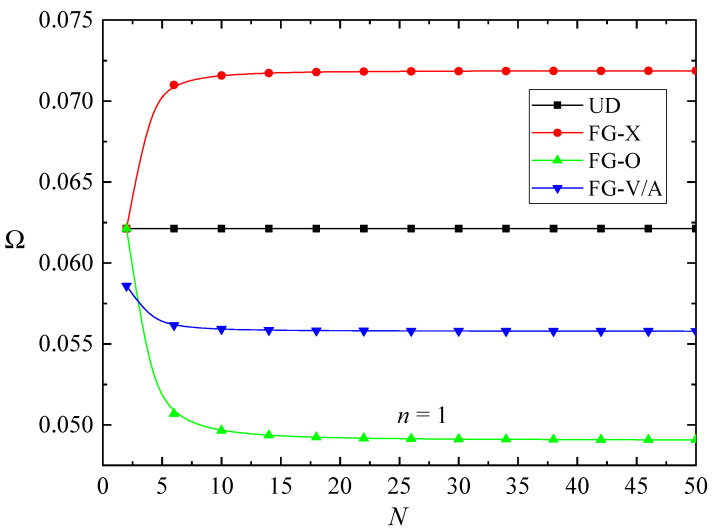
The effects of the total layer number *N* on the fundamental frequency **Ω** of the FG-GPLRC beams’ absence of magnetic fields and elastic foundation.

Figure 3 plots the effects of the total layer number *N* of the FG-GPLRC beam (λ=1/10) on the dimensionless fundamental frequency Ω for different GPL distribution patterns. For the UD pattern, the vibration frequencies are independent of the total number *N* due to the uniform distribution of GPLs. However, the total layer number *N* plays a critical role in the vibration frequencies of the patterns with GPLs dispersed nonuniformly. For a fixed total volume fraction *W*_GPL_, with the increasing total layer number *N*, the fundamental frequencies increase distinctly first and then vary slightly for the FG-X pattern. However, the fundamental frequencies decrease significantly first and then remain nearly unchanged for the FG-O and FG-V/A patterns. In the FG-X pattern, more GPLs are dispersed near the top and bottom layers with the increasing total layer number *N*, which is more powerful for promoting stiffness and hence increases the vibration frequencies of the beam. Moreover, it can be concluded that the multilayer beam with the sufficiently large number of individual layers is an excellent alternative for the functionally graded GPL-reinforced nanocomposites. In the following analysis, the total layer number *N* = 20 is adopted.

Table 4 gives the first-five order vibration frequencies Ω of FG-GPLRC beams without considering the effects of magnetic fields and elastic foundation. The corresponding vibration frequencies of pure epoxy beams are involved for comparisons. It can be seen that, regardless of GPL distribution patterns, the vibration frequencies of the beams increase significantly even by adding a low content of GPLs into the epoxy matrix. As expected, the vibration frequencies of FG-V and FG-A are identical when neglecting the effects of an elastic foundation. The FG-X GPLRC beam has the largest, while FG-O GPLRC beam has the lowest fundamental frequencies among the five beams. In the FG-X pattern, more GPLs are dispersed near the top and bottom surfaces of the beam where the normal stresses are higher. Therefore, the GPL nanofillers can maximize the reinforcing effects to increase the stiffness of the beam.

Table 5 demonstrates the effects of the elastic foundation on the fundamental frequencies Ω of the FG-GPLRC beams in absence of magnetic fields. It is obvious that both the Winkler and shearing layer elastic coefficients have significant effects on the fundamental frequency parameters. All the fundamental frequencies increase dramatically when promoting the elastic coefficients of the foundation. However, for the large values of the Winkler elastic coefficient, the shearing layer elastic coefficient has less effect on the fundamental frequencies. The effects of the GPL distribution pattern are also listed in the table. It was found again that the distribution pattern of GPLs can critically influence the fundamental frequencies. The vibration frequencies of FG-A and FG-V are quite different when considering the elastic foundation. In the FG-A pattern, the bottom surface of the beam is GPL-rich, and the fundamental frequency is higher than that of FG-A. Moreover, among all the GPL patterns, the FG-X pattern holds the highest fundamental frequencies. It can be concluded that the FG-X pattern can sufficiently utilize the reinforcing effects of GPLs and increase the bending stiffness of the FG-GPLRC beams more powerfully.

The effects of the magnetic fields along different directions on the fundamental frequencies are tabulated in Table 6. It can be observed that the magnetic field significantly influences the fundamental frequencies of the FG-GPLRC beam. The fundamental frequencies of the FG-GPLRC beam increase as we increase the values of the magnetic fields both along the *x* and *y* directions. However, the fundamental frequencies decrease as the *z*-direction magnetic field is increased. As shown in Equation (10), the *x*-direction magnetic field induces the transverse Lorentz force through the thickness direction of the beam, and the *y*- and *z*-direction magnetic fields induce the longitudinal Lorentz forces, respectively. In addition, the Lorentz force induced by the *z*-direction magnetic fields is only decided by the longitudinal displacement *u*. However, the Lorentz forces induced by the other two-direction magnetic fields are still concerned with the transverse displacement *w*. Therefore, the trend of the *z*-direction magnetic field on the fundamental frequencies differs from the others. It should be noted that the fundamental frequencies of the beam change slightly in a strong *x*-direction magnetic field (larger value of *M_x_*). Regardless of the magnetic fields, the addition of GPLs into the matrix can improve the stiffness of the beams and hence promote the corresponding vibration frequencies. Moreover, the FG-X pattern gives the highest fundamental frequencies in all magnetic fields, and this will be focused on in the following discussion.

**Figure 4:**
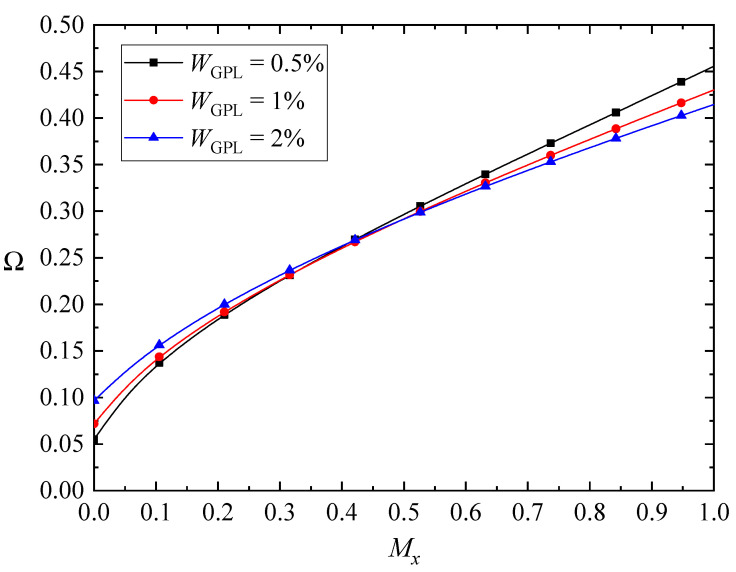
Variation of the fundamental frequency Ω with the *x*-direction magnetic field (λ = 1/10, *K_w_* = *K_p_* = 0).

**Figure 5:**
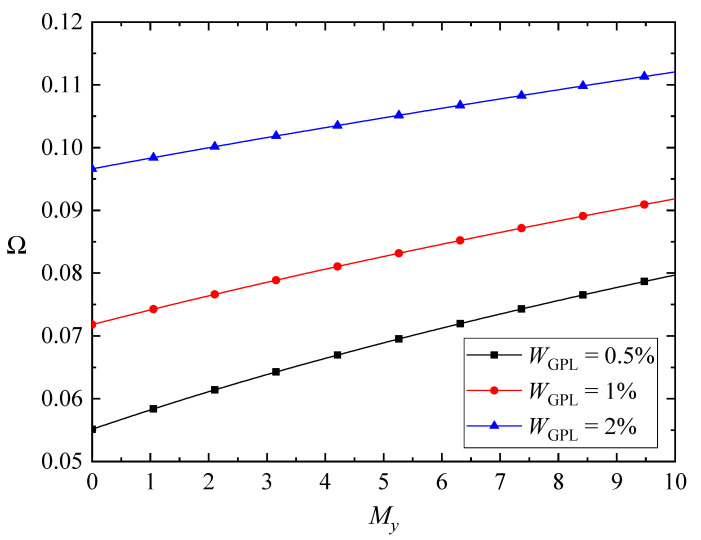
Variation of the fundamental frequency Ω with the *y*-direction magnetic field (λ = 1/10, *K_w_* = *K_p_* = 0).

**Figure 6:**
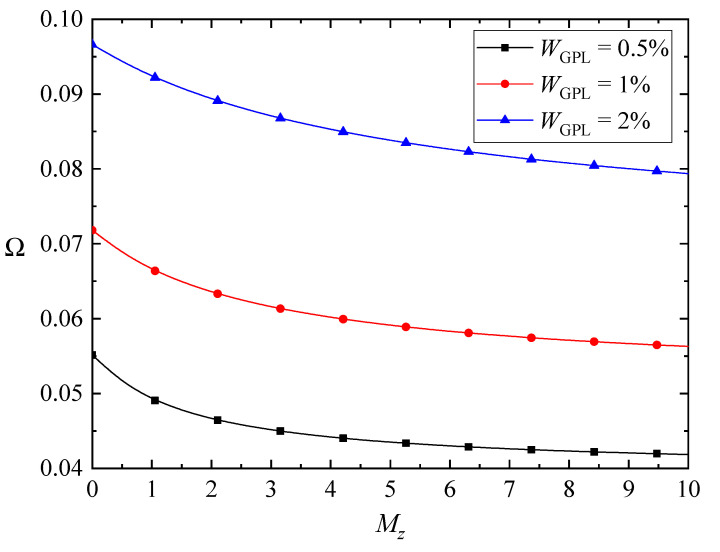
Variation of the fundamental frequency Ω with the *z*-direction magnetic field (λ = 1/10, *K_w_* = *K_p_* = 0).

The variations of the fundamental frequencies of the FG-GPLRC beams with various magnetic parameters as well as the GPL weight fraction are plotted in Figure 4, Figure 5 and Figure 6. Here, only the weak magnetic field in the *x* direction is involved in Figure 4. The fundamental frequencies of the beams increase with the promotion of magnetic fields, except for the case of the beams in the *z*-direction magnetic field. As the GPLs are more dispersed, the fundamental frequencies of the beams increase for the *y*-direction and *z*-direction magnetic fields, while the fundamental frequencies of the beams in the *x*-direction magnetic field increase first and then decrease. As stated before, the *x*-direction magnetic field induces only the *z*-direction Lorentz force, and the corresponding Lorentz force is determined by the magnetic field and the bending deformations. The transverse displacement of the beam decreases when adding more GPL nanofillers into the matrix, while the competition between the bending deformation and the magnetic field leads to the complicated variation of the fundamental frequencies.

**Figure 7:**
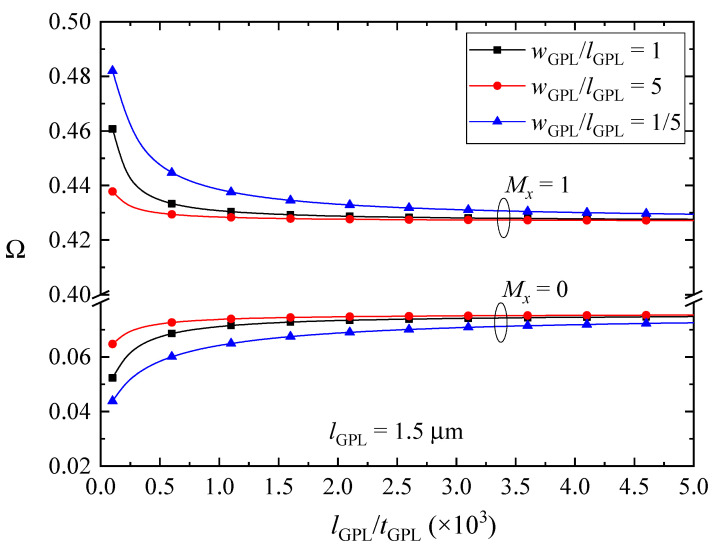
Effects of size and geometric configuration of GPLs on the fundamental frequency Ω of the beams in the *x*-direction magnetic field (λ = 1/10, *K_w_* = *K_p_* = 0).

**Figure 8:**
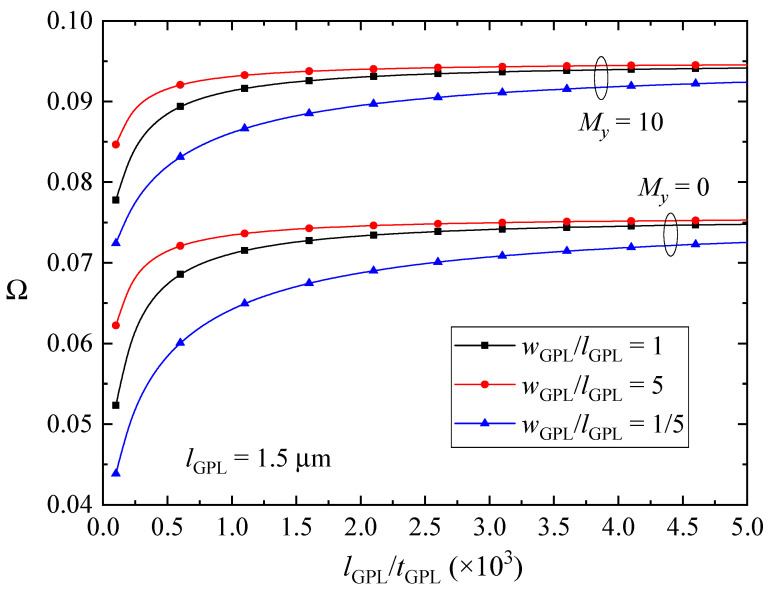
Effects of size and geometric configuration of GPLs on the fundamental frequency Ω of the beams in the y-direction magnetic field (λ = 1/10, *K_w_* = *K_p_* = 0).

**Figure 9:**
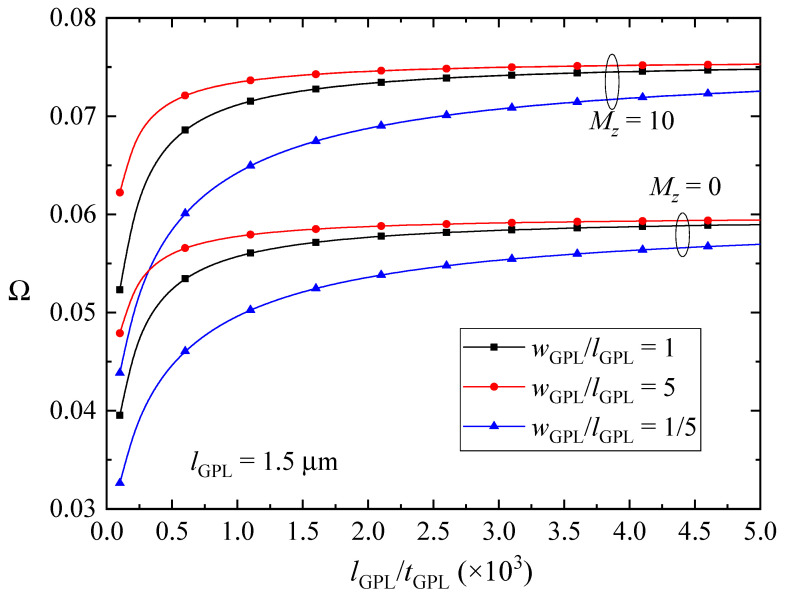
Effects of size and geometry of GPLs on the fundamental frequency Ω of the beams in the *z*-direction magnetic field (λ = 1/10, *K_w_* = *K_p_* = 0).

The effects of size and geometry of GPL nanofillers on the fundamental frequencies of the FG-GPLRC beams with various magnetic parameters are depicted in Figure 7, Figure 8 and Figure 9. The fundamental frequencies of the beams increase sharply and then change slightly with decreasing the thickness of the GPL nanofillers when the beams are absent of the magnetic fields or in *y*-direction and *z*-direction magnetic fields. The GPLs as nanofillers dispersed into the epoxy matrix can dramatically improve the bending stiffness of the beams, and thus the vibration frequencies will be promoted as expectedly. Moreover, thinner GPL nanofillers can increase the stiffness of the beams more powerfully. The magnetic fields applied along the *y* direction and *z* direction induce the Lorentz forces in the *x* direction, which can be evaluated by the longitudinal and transverse displacements as shown in Equation (10) and can help to increase the stiffness of the beams. However, the fundamental frequencies of the FG-GPLRC beams in the *x*-direction magnetic field reduce when the GPL nanofillers are thinner. It is also the result of the competition between the bending deformation and the magnetic field. The thinner GPL nanofillers can powerfully increase the bending stiffness of the beams, and hence the transverse displacements become smaller and smaller. Accordingly, the correlative displacement terms in Equation (18) and the corresponding fundamental frequencies decrease. It is also can be observed that GPL nanofillers with larger surfaces can increase the stiffness more efficiently. This is due to the fact that larger surfaces between the GPL nanofillers and the matrix can transfer loads better. It can be concluded that thinner and larger GPL nanofillers are preferred as nano-reinforcements for increasing the fundamental frequencies of FGGPLRC beams; however, it reverses for the case of the beams in the *x*-direction magnetic field.

**Figure 10:**
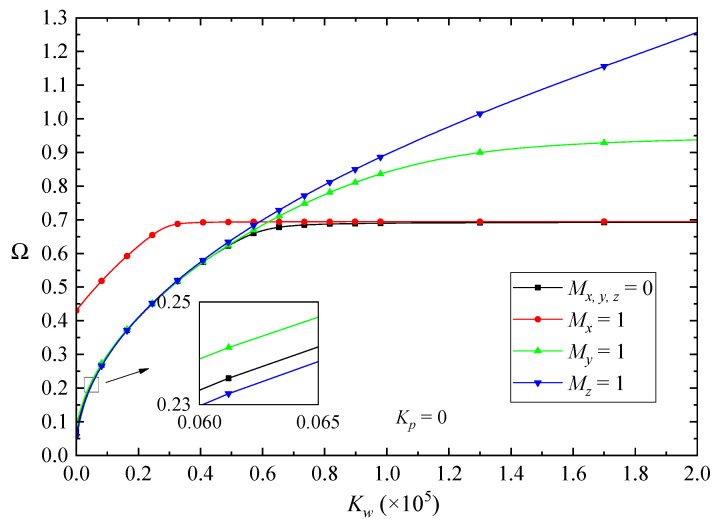
Effects of the Winkler coefficient on the fundamental frequency Ω of the beams in magnetic fields (λ = 1/10).

**Figure 11:**
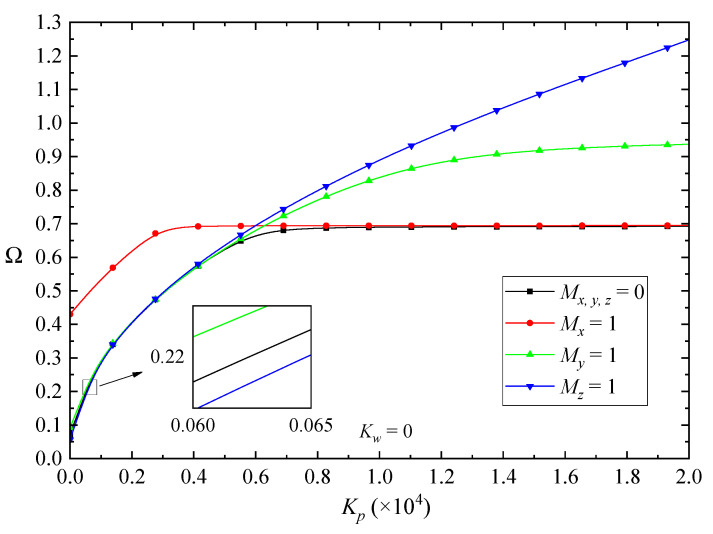
Effects of the Pasternak coefficient on the fundamental frequency Ω of the beams in magnetic fields (λ = 1/10).

Figure 10 and Figure 11 show the effects of the Winkler and Pasternak coefficients of the elastic foundation on the fundamental frequencies of the FG-GPLRC beams, respectively. According to the figures, by increasing the Winkler or Pasternak coefficients, all vibration frequencies increase first and then almost keep unchanged. Moreover, it was observed that the magnetic field has a critical effect on the fundamental frequencies of the beams. In lower values of the Winkler/Pasternak coefficient, the *x*-direction magnetic field promotes the vibration frequencies more remarkably than that in *y*-direction magnetic field. On the contrary, the z-direction magnetic field decreases the fundamental frequencies. However, in higher values of the Winkler/Pasternak coefficient, the fundamental frequencies of the beams nearly keep constant when increasing the elastic coefficients of the foundation. The effects of the *x*-direction magnetic field are neglectable, while the magnetic field along the *z*-direction increases the vibration frequencies more dramatically.

## 4. Conclusions

This article presented the frequency analysis of the FG-GPLRC beams resting on an elastic foundation. The equations of motion, Maxwell’s equation, and simply supported boundary conditions were derived using the state space method based on a two-dimensional elasticity theory. The frequency equation was derived analytically by using the interface continuum conditions. Furthermore, for confirmation, the results of the current method were validated with the results obtained from other references. Finally, in the results section the effects of the elastic foundation, magnetic fields, as well as the distribution pattern, weight function, size, and geometry of GPL nanofillers on the frequency characteristics of the FG-GPLRC beams were studied. In this study, the following main results were achieved:

(1) A low content of GPLs dispersed into the matrix can increase the vibration frequencies significantly, and the pattern FG-X holds the highest fundamental frequency among all GPL distribution patterns.
(2) The results show that an increase in the elastic coefficients of the elastic foundation promotes the frequency characteristics of the FG-GPLRC beams.
(3) The results show that an increase in the magnetic fields of the *x* direction and *y* direction increases the fundamental frequencies of the FG-GPLRC beams. However, it reverses for the case of *z*-direction magnetic field.
(4) Thinner and larger GPL nanofillers are preferred as nano-reinforcements to increase the fundamental frequencies of FG-GPLRC beams in the *y*- and *z*-direction magnetic fields. However, it reverses for the case of the beams in *x*-direction magnetic field.

## Figures and Tables

**Table 1:** Comparisons of the fundamental frequency parameter ϖ of an isotropic homogeneous beam on elastic foundations.

$K_{w}$	$K_{p}/\pi^{2}$	λ=1/120		λ=1/15		λ=1/5	
		Present	Reference [48]	Present	Reference [48]	Present	Reference [48]
0	0.0	3.14143	3.14143	3.13024	3.13024	3.04799	3.04799
	0.5	3.47659	3.47659	3.46670	3.46671	3.39458	3.39458
	1.0	3.73587	3.73587	3.72656	3.72656	3.65801	3.65802
	2.5	4.29686	4.29686	4.28808	4.28809	4.21833	4.21834
10^2^	0.0	3.74823	3.74823	3.73894	3.73894	3.67049	3.67050
	0.5	3.96067	3.96067	3.95167	3.95168	3.88397	3.88397
	1.0	4.14356	4.14356	4.13471	4.13471	4.06635	4.06636
	2.5	4.58226	4.58226	4.57346	4.57347	4.49913	4.49913
10^4^	0.0	10.02403	10.02403	9.99581	9.99582	7.34080	7.34081
	0.5	10.03610	10.03610	10.00777	10.00778	7.34087	7.34088
	1.0	10.04813	10.04813	10.01968	10.01969	7.34094	7.34095
	2.5	10.08394	10.08394	10.05518	10.05519	7.34115	7.34116

**Table 2:** Comparisons of the fundamental frequency parameter ϖ of an FGM beam on elastic foundations.

$K_{w}$	$K_{p}$	λ=1/15		λ=1/5		λ=1/2.5	
		Present	Reference [49]	Present	Reference [49]	Present	Reference [49]
0	0	2.9449	2.9449	2.8773	2.8773	2.7026	2.7026
	10	3.1644	3.1644	3.0979	3.0979	2.9194	2.9194
	25	3.4264	3.4264	3.3578	3.3578	3.1450	3.1450
102	0	3.1670	3.1670	3.1005	3.1005	2.9219	2.9219
	10	3.3480	3.3480	3.2804	3.2804	3.0809	3.0809
	25	3.5743	3.5743	3.5030	3.5030	3.2583	3.2583
104	0	7.1422	7.1422	6.5058	6.5058	4.2742	4.2742
	10	7.1592	7.1592	6.5163	6.5163	4.2753	4.2753
	25	7.1844	7.1844	6.5319	6.5319	4.2770	4.2770

**Table 3:** Material properties of the GPLs and epoxy [7].

Material Properties	GPLs	Epoxy
Young’s modulus (GPa)	1010	3.0
Density (kg/m^3^)	1060	1200
Poisson’s ratio	0.186	0.34

**Table 4:** Frequency parameter Ω of FG-GPLRC beams’ absence of magnetic fields and elastic foundation.

*λ*	*n*	Epoxy	UD	FG-X	FG-O	FG-V	FG-A
1/20	1	0.0075	0.0157 (109%)	0.0184 (145%)	0.0124 (65%)	0.0141 (88%)	0.0141 (88%)
	2	0.0298	0.0621 (108%)	0.0718 (141%)	0.0491 (65%)	0.0558 (87%)	0.0558 (87%)
	3	0.0656	0.1369 (109%)	0.1554 (137%)	0.1091 (66%)	0.1233 (88%)	0.1233 (88%)
	4	0.1134	0.2367 (109%)	0.2628 (132%)	0.1906 (68%)	0.2140 (89%)	0.2140 (89%)
	5	0.1714	0.3579 (109%)	0.3882 (126%)	0.2915 (70%)	0.3249 (90%)	0.3249 (90%)
1/10	1	0.0298	0.0621 (108%)	0.0718 (141%)	0.0491 (65%)	0.0558 (87%)	0.0558 (87%)
	2	0.1134	0.2367 (109%)	0.2628 (132%)	0.1906 (68%)	0.2140 (89%)	0.2140 (89%)
	3	0.2380	0.4968 (109%)	0.5266 (121%)	0.4097 (72%)	0.4530 (90%)	0.4530 (90%)
	4	0.3903	0.8149 (109%)	0.8283 (112%)	0.6890 (77%)	0.7502 (92%)	0.7502 (92%)
	5	0.5602	1.1699 (109%)	1.1491 (105%)	1.0131 (81%)	1.0871 (94%)	1.0871 (94%)
1/5	1	0.1134	0.2367 (109%)	0.2628 (132%)	0.1906 (68%)	0.2140 (89%)	0.2140 (89%)
	2	0.3903	0.8149 (109%)	0.8283 (112%)	0.6890 (77%)	0.7502 (92%)	0.7502 (92%)
	3	0.7409	1.5474 (109%)	1.4803 (100%)	1.3694 (85%)	1.4507 (96%)	1.4507 (96%)
	4	1.1191	2.3376 (109%)	2.1622 (93%)	2.1452 (92%)	2.2242 (99%)	2.2242 (99%)
	5	1.5060	3.1461 (109%)	2.8653 (90%)	2.9689 (97%)	3.0225 (101%)	3.0225 (101%)

**Table 5:** The effects of an elastic foundation on the fundamental frequency Ω of FG-GPLRC beams (*λ* = 1/10, *M_x_* = *M_y_* = *M_z_* = 0).

*K_w_*	*K_p_*	Epoxy	UD	FG-X	FG-O	FG-V	FG-A
0	0	0.0298	0.0621	0.0718	0.0492	0.0558	0.0558
	10	0.0412	0.0683	0.0772	0.0568	0.0626	0.0627
	50	0.0702	0.0889	0.0960	0.0804	0.0845	0.0848
	100	0.0947	0.1094	0.1152	0.1026	0.1056	0.1061
10^2^	0	0.0413	0.0684	0.0773	0.0569	0.0627	0.0628
	10	0.0501	0.0741	0.0824	0.0637	0.0688	0.0690
	50	0.0758	0.0934	0.1002	0.0854	0.0891	0.0895
	100	0.0989	0.1131	0.1187	0.1065	0.1094	0.1099
10^3^	0	0.0952	0.1099	0.1156	0.1031	0.1061	0.1066
	10	0.0994	0.1135	0.1191	0.1069	0.1098	0.1104
	50	0.1144	0.1269	0.1320	0.1211	0.1235	0.1242
	100	0.1308	0.1420	0.1465	0.1368	0.1388	0.1396
10^5^	0	0.3302	0.4076	0.4091	0.4050	0.4026	0.4086
	10	0.3303	0.4086	0.4100	0.4060	0.4036	0.4095
	50	0.3304	0.4125	0.4139	0.4098	0.4073	0.4134
	100	0.3324	0.6910	0.6898	0.6908	0.6818	0.6936

**Table 6:** The magnetic effects on the fundamental frequency Ω of FG-GPLRC beams (λ = 1/10, *K_w_* = *K_p_* = 0).

Magnetic Parameter		Epoxy	UD	FG-X	FG-O	FG-V	FG-A
*M_x_*	0	0.0298	0.0621	0.0718	0.0492	0.0558	0.0558
	2	0.3351	0.6481	0.6959	0.6960	0.6878	0.6878
	5	0.3371	0.6983	0.6977	0.6980	0.6964	0.6964
	10	0.3400	0.6999	0.6997	0.6993	0.6982	0.6982
*M_y_*	0	0.0298	0.0621	0.0718	0.0492	0.0558	0.0558
	2	0.0401	0.0678	0.0764	0.0564	0.0631	0.0631
	5	0.0513	0.0754	0.0827	0.0656	0.0721	0.0721
	10	0.0649	0.0864	0.0918	0.0784	0.0843	0.0843
*M_z_*	0	0.0298	0.0621	0.0718	0.0492	0.0558	0.0558
	2	0.0228	0.0532	0.0636	0.0409	0.0471	0.0471
	5	0.0220	0.0495	0.0591	0.0382	0.0441	0.0441
	10	0.0216	0.0474	0.0563	0.0369	0.0424	0.0424
